# Validation of the Arabic version of the asthma control test

**DOI:** 10.4103/1817-1737.39635

**Published:** 2008

**Authors:** H. Lababidi, A. Hijaoui, M. Zarzour

**Affiliations:** *Department of Critical Care, King Fahad Medical City, Riyadh, Saudi Arabia*; 1 *Department of Internal Medicine, Makassed General Hospital, Beirut, Lebanon*

**Keywords:** Asthma, asthma assessment, asthma control test

## Abstract

**PURPOSE:**

Asthma control test (ACT) has been devised to assess the degree of asthma control in out-patients setting. The aim of this study is to validate the Arabic version of ACT.

**MATERIALS AND METHODS:**

Patients completed the Arabic version of ACT during regular visit to one of two asthma specialists. Spirometry was obtained. The asthma specialist rated asthma control using a 5-point scale and indicated modification in management as step up, same or step down of asthma treatment.

**RESULTS:**

40 patients completed the study, the mean age was 32.6 + 14.0 years, mean FEV1 was 2.7 + 1.0 L (89.2% + 23.6% of predicted). The mean ACT score was 15.9 + 5.8; mean of specialist asthma control rating was 3.4 + 1.0. The internal consistency reliability of the 5-item ACT survey was alpha = 0.92. The correlation was moderate between ACT and specialists rating (*r* = 0.482, *P* = 0.002) and between ACT and treatment modification (*r* = −0.350, *P* = 0.027). The correlation between FEV1 and ACT was low (*r* = 0.185, *P* = 0.259). ACT distinguished between patients with different specialist rating (*F* = 3.37, *P* = 0.02) and the need to change therapy (*F* = 3.62, *P* = 0.037). The areas under the curve (ROC) for ACT, FEV1, and ACT and FEV1 as independent variables were 0.720, 0.721, and 0.766 respectively. All results were comparable to the initial work for development of ACT.

**CONCLUSION:**

The Arabic version of the ACT is a valid tool to assess asthma control. ACT correlates better with asthma specialist rating of asthma control than with FEV1.

Asthma is a clinical diagnosis made by physicians on the basis of patient's medical history, physical examination, assessment of the reversibility of airway obstruction, and exclusion of alternative diagnosis that mimic asthma.[1] Asthma is a very common disease with immense social impact. The global prevalence of asthma ranges from 1% to 18%.[2] The improved understanding of the pathophysiology underlying asthma and the emergence of medications to prevent acute exacerbations more effectively have led clinicians to shift their focus from managing acute attack to achieving asthma control.[3] According to international guidelines, the primary goal of asthma management is to achieve and maintain asthma control defined as ‘no daytime symptoms, no limitations of daily activities, no nocturnal symptoms or awakening, no need for reliever treatment, normal or near-normal lung function results and no exacerbations.’[4] Based on results of large multinational, community-based surveys of asthma showing that the majority of patients have an alarmingly high rate of symptoms and disruption of life from their disease,[5] one can say that this goal has not been achieved.

Asthma control can be difficult to assess in clinical practice because of its multidimensional nature and variability over time. Tools that are easily and quickly administered in clinical practice are required in order to develop an accurate, quick, and practical asthma control test. Thus, the need for a simple method for quantifying asthma control by both patients and physicians has brought up the development of a short assessment tool called the asthma control test (ACT). This tool is a five-item questionnaire developed as an easy method for patients and clinicians to assess symptoms, use of rescue medications, and impact on activities.[3] Each question is graded from one to five. The score range is 5 to 25. Well-controlled asthma is defined as a score above 20 on ACT. Asthma control test has been translated into different languages. The Arabic version has been professionally translated and distributed by the pharmaceutical company Glaxo Smith Kline (GSK).[6]

To the best of our knowledge, the Arabic version of ACT has not been clinically validated yet. We elected to replicate the original study of the ACT by Nathan *et al.*,[3] but with the Arabic version. Similar results will indicate validation of the Arabic version of the ACT. The aim of this study is to validate the Arabic version of ACT with regard to FEV_1_ and asthma control as assessed by asthma specialists.

## Materials and Methods

This is a prospective study performed over a 3-month period, from November 2006 to January 2007. Patients older than 10 years who had been diagnosed with asthma and who were literate in Arabic were eligible for participation unless they had other respiratory conditions or were participating in other clinical studies. Patients were recruited from two asthma specialists’ practices. Institutional Review Board at Makassed General Hospital approved the study, and all patients or guardians signed a written informed consent. Oral approval to use the Arabic version of ACT was obtained from GSK, Lebanon.

Participants completed the ACT during a routine physician office visit before they were seen by their physician. After the patient completed the survey, pre-bronchodilator measurements of FEV_1_ were obtained. The physician, who was blinded to each subject's survey responses, interviewed the patient. During the interview, the level of asthma control for each subject was rated by the asthma specialist on a five-point scale ranging from ‘not controlled at all’ to ‘completely controlled.’ This rating of asthma control was based on how well the goals of asthma therapy were being met, as outlined in the GINA guidelines[4] and from the history and physical examination. The rating of asthma control was applied across all asthma severity levels. The asthma specialist also indicated on a special form the modification of treatment of asthma as ‘stepped down,’ ‘no change,’ or ‘stepped up.’ In addition, age, gender, age of asthma symptoms, onset, family history of asthmatic patients were recorded.

### Statistical analysis

The analysis of internal consistency reliability of the five-item ACT was conducted among all the patients recruited for the study. ANOVA testing was used to compare ACT means across groups differing in asthma control with specialists’ rating of control and with treatment modification; a *P*-value of < 0.05 was considered significant.

Receiver operating characteristic (ROC) analysis was conducted to compare and contrast the ability of the independent variables to screen for subjects with poorly controlled asthma. The accuracy of the ACT depends on how well it can separate the group being tested into those with and without asthma control. Accuracy is measured by the area under the ROC curve. An area of 1 represents a perfect test; an area of 0.5 represents a worthless test.

Nonparametric Spearman's correlation coefficient was conducted between ACT score and specialist's rating ACT score and treatment modification, a *P*-value of < 0.05 was considered significant.

## Results

Forty patients were included in this study. The mean age was 32 ± 14 years, the age of onset of asthma was 13 ± 15 years; there were 15 males (37.5%) and 25 females (62.5%). Family history of asthma was present in 23 patients (57.5%). The mean FEV_1_ was 2.7 ± 1.0 L, and the mean ACT score was 15.9 ± 5.8. The mean of physicians' asthma control rating was 3.4 ± 1.0. The internal consistency reliability Alpha was 0.92, indicating a high consistency among the answers to the five different questions of the ACT.

The correlation between means of ACT scores and specialists’ ratings of asthma control and treatment modification is presented in Tables 1 and 2. Both results were statistically significant, with F = 3.4 (*P* = 0.02) and F = 3.6 (*P* = 0.037) respectively.

**Table 1:** Comparison between asthma control test scores and asthma specialists’ ratings of control

Specialist rating of control							

	Not controlled at all (n = 2)	Poorly controlled (n = 5)	Somewhat controlled (n = 15)	Well controlled (n = 12)	Completely controlled (n = 6)	F	*P*-value
ACT score	8.5 ± 0.7	13.6 ± 3.9	14.9 ± 5.6	16.6 ± 5.6	21.8 ± 4.7	3.4	0.02

**Table 2:** Comparison between asthma control test score and asthma treatment modification

Asthma treatment modification					

	Stepped down (n = 3)	No change (n = 19)	Stepped up (n = 18)	F	*P*-value
ACT score	23.3 ± 2.9	16.4 ± 5.7	14.3 ± 5.5	3.6	0.037

**Figure 1:**
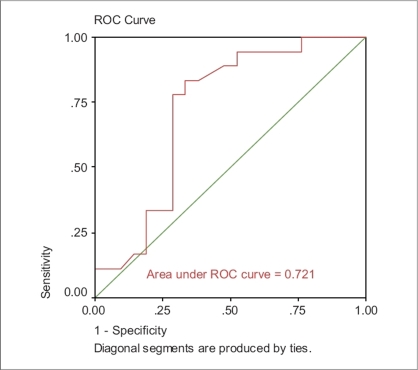
FEV_1_ as independent variable

**Figure 2:**
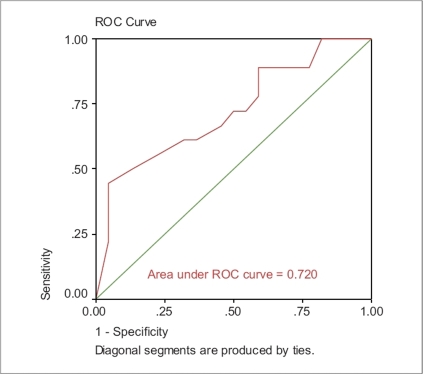
ACT as independent variable

**Figure 3:**
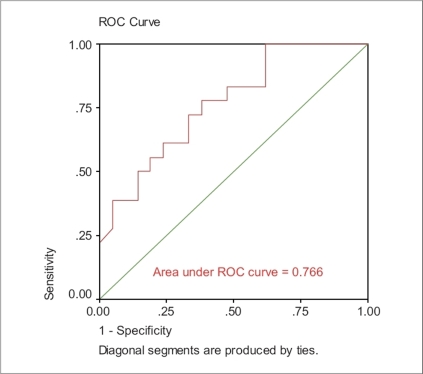
ACT + FEV_1_ as independent variable

The area under ROC for ACT score was 0.720, whereas the area under ROC for FEV_1_ was 0.721 [Figures 1 and 2]. Moreover, the area under ROC for both variables (ACT score and FEV_1_) was 0.766 [Figure 3].

There was a significant correlation of r = 0.482 between ACT score and specialist’s rating (*P* = 0.002). In addition, a significant correlation of r = −0.350 also exists between the ACT score and treatment modification (*P* = 0.027). However, the correlation between FEV_1_ and ACT did not reach a significant level (r = 0.185, *P* = 0.259).

## Discussion

The previous asthma severity classification into intermittent, mild persistent, moderate persistent or severe persistent is no more recommended. Instead, current guidelines focus more on levels of asthma control and suggest subdividing asthma into controlled, partly controlled, and uncontrolled.[4] Validated tools have been developed to measure asthma control, such as asthma control questionnaire (ACQ),[5] asthma control scoring system,[7] asthma therapy assessment questionnaire (ATAQ),[8] and asthma control test (ACT).[3] The ACT is a short, easy-to-use, and reliable measure of asthma control.[9]

The main finding of this survey is the similarity in the results of validation between the original work for the development of ACT[3] and our work, indicating a strong validation of the Arabic version of the ACT. The internal reliability of the five questions in the ACT survey was 0.92 in our sample, which is very similar to that obtained by Nathan *et al.*, 0.84, in their development of the ACT through a survey based on a substantially representative sample.[3]

Moderate correlations between the ACT on one hand and the specialists’ rating and the treatment modification on the other hand were observed. The highest correlation was observed between the specialist’s rating and ACT score (r = 0.48, *P* = 0.002). These findings are in accordance with those obtained in the original study to validate ACT score.[3]

In the main study,[3] there was a significant correlation between FEV_1_ and ACT score (r = 0.19, *P* = 0.0001). In our study, the correlation between FEV_1_ and ACT was not statistically significant (r = 0.18, *P* = 0.259). This could be explained by the small sample size (*n* = 40) in comparison to the original sample, 436 patients. The utility of FEV_1_ to reflect asthma control has been questionable, in previous studies. Moy *et al.* found no significant correlation between FEV_1_ and health-related quality of life (HRQL) questionnaire in patients with asthma.[10] Poor correlation was found between asthma rate control and pulmonary function, even in patients with mild-to-moderate uncontrolled asthma in another survey done by Boulet *et al.*[7]

ACT score provides a greater predictive value in determining the patient's asthma control than does a predicted % FEV_1_ value. However, the best measure of control would be a combination of both ACT score and FEV_1_. Our ROC analysis was very similar to the analyses reported by Nathan *et al.* (0.720 and 0.766).[3] The limitation of this study lies in the small sample size.

In conclusion, the Arabic version of the ACT is a valid tool for Arabic-speaking patients to assess asthma control. ACT score correlates better with asthma specialist's rating of asthma control than with FEV_1_.

## References

[1] Eder W, Ege MJ, von Mutius E (2006). The asthma epidemic. N Engl J Med.

[2] Masoli M, Fabian D, Holt S, Beasley R (2004). The global burden of asthma: Executive summary of the GINA Dissemination Committee report. Allergy.

[3] Nathan RA, Sorkness CA, Kosinski M, Schatz M, Li JT, Marcus P (2004). Development of the asthma control test: A survey for assessing asthma control. J Allergy Clin Immunol.

[4] Global Strategy for Asthma Management and Prevention 2006.

[5] Juniper EF, Buist AS, Cox FM, Ferrie PJ, King DR (1999). Validation of a standardized version of the Asthma Quality of Life Questionnaire. Chest.

[6] Bateman ED, Boushey HA, Bousquet J, Busse WW, Clark TJ, Pauwels RA (2004). Can guideline-defined asthma control be achieved? The Gaining Optimal Asthma Control study. Am J Respir Crit Care Med.

[7] Boulet LP, Boulet V, Milot J (2002). How should we quantify asthma control? A proposal. Chest.

[8] Vollmer WM, Markson LE, O'Connor E, Sanocki LL, Fitterman L, Berger M (1999). Association of asthma control with health care utilization and quality of life. Am J Respir Crit Care Med.

[9] Schatz M, Sorkness CA, Li JT, Marcus P, Murray JJ, Nathan RA, Kosinski M (2006). Asthma Control Test: Reliability, validity and responsiveness in patients not previously followed by asthma specialists. J Allergy Clin Immunol.

[10] Moy ML, Israel E, Weiss ST, Juniper EF, Dubé L, Drazen JM (2001). Clinical predictors of health-related quality of life depend on asthma severity. Am J Respir Crit Care Med.

